# Genome-wide identification of signaling center enhancers in the developing limb

**DOI:** 10.1242/dev.110965

**Published:** 2014-11

**Authors:** Julia E. VanderMeer, Robin P. Smith, Stacy L. Jones, Nadav Ahituv

**Affiliations:** 1 Department of Bioengineering and Therapeutic Sciences, University of California San Francisco, San Francisco, CA 94158, USA; 2 Institute for Human Genetics, University of California San Francisco, San Francisco, CA 94158, USA

**Keywords:** Enhancer, AER, ZPA, Limb, Mouse

## Abstract

The limb is widely used as a model developmental system and changes to gene expression patterns in its signaling centers, notably the zone of polarizing activity (ZPA) and the apical ectodermal ridge (AER), are known to cause limb malformations and evolutionary differences in limb morphology. Although several genes that define these limb signaling centers have been described, the identification of regulatory elements that are active within these centers has been limited. By dissecting mouse E11.5 limbs that fluorescently mark the ZPA or AER, followed by fluorescence-activated cell sorting and low-cell H3K27ac ChIP-seq, we identified thousands of specific signaling-center enhancers. Our ChIP-seq datasets show strong correlation with ZPA- and AER-expressed genes, previously characterized functional ZPA and AER enhancers and enrichment for relevant biological terms related to limb development and malformation for the neighboring genes. Using transgenic assays, we show that several of these sequences function as ZPA and AER enhancers. Our results identify novel ZPA and AER enhancers that could be important regulators of genes involved in the establishment of these specialized regions and the patterning of tetrapod limbs.

## INTRODUCTION

The tetrapod limb has long been used as a model of embryonic development in three dimensions. The limb bud begins as a small protrusion of cells from the flank of the embryo. As it develops, the limb bud must establish polarity along three axes: anterior-posterior (AP), proximal-distal (PD) and dorsal-ventral (DV). The AP axis is controlled by a small region in the posterior limb bud called the zone of polarizing activity (ZPA). These cells express *sonic hedgehog* (*Shh*), which acts as a morphogen to determine the AP axis. When *Shh* is expressed ectopically in anterior parts of the limb bud, the pattern of the AP axis is disrupted. The most common phenotype caused by anterior ‘ZPA activity’ is the formation of extra digits at the anterior of the limb or the adoption of posterior identity by anterior digits (reviewed by Anderson et al. (2012)]. The PD axis is maintained by the apical ectodermal ridge (AER), which develops from the ectoderm at the distal edge of the limb bud. Without a functioning AER, the growth of the limb is severely stunted. There is significant crosstalk between the ZPA and AER, with each signaling center required for the maintenance of the other [reviewed by Zeller et al. (2009)].

Gene expression changes in signaling centers are known to affect limb morphology. As the genes involved in the ZPA and AER are also expressed in other tissues, mutations in coding regions usually cause additional phenotypes. Mutations in enhancers that are specific to these limb-signaling centers, however, only cause limb phenotypes. For example, mutations in the ZPA regulatory sequence (ZRS) enhancer that controls expression of *Shh* in the ZPA are known to cause isolated limb malformations in humans, mice, cats and dogs, without any other phenotypes caused by coding disruptions to *Shh* [reviewed by VanderMeer and Ahituv (2011)]. Another study showed that replacing the mouse *Prx1* limb enhancer with the bat homologous enhancer leads to longer bones in the mature limb, but no other phenotypes (Cretekos et al., 2008).

The identification of enhancers in the ZPA and AER is challenging. Cells in these signaling centers form only ∼4% of the developing limb. Whereas previous studies have used chromatin immunoprecipitation followed by sequencing (ChIP-seq) on histone marks (Cotney et al., 2012, 2013) or E1A binding protein p300 (EP300/p300) (Visel et al., 2009), these address the limb as a single tissue. Because the ZPA and AER have functions distinct from the larger limb mesenchyme, it is likely that they have distinct epigenetic signatures that would be diluted in such an experiment. Tellingly, not a single one of the twenty p300 ChIP-seq peaks that were positive for limb enhancer activity (Visel et al., 2009) were expressed specifically in the ZPA or AER.

To identify active enhancers specific to signaling centers, it is necessary to study these tissues independently. We therefore bred mouse lines that endogenously express GFP in either the ZPA or AER to isolate the cells of each signaling center using fluorescence-activated cell sorting (FACS). We then used ChIP-seq to identify regions with the epigenetic signature H3K27ac, which is associated with active enhancers (Creyghton et al., 2010; Rada-Iglesias et al., 2012). Subsequent mouse transgenic enhancer assays on selected ZPA and AER ChIP-seq peaks found them to be active in these signaling centers. Through this work we have identified two novel sets of signaling center-specific enhancers which can play important roles in limb development and morphology.

## RESULTS

**Fig. 1.**
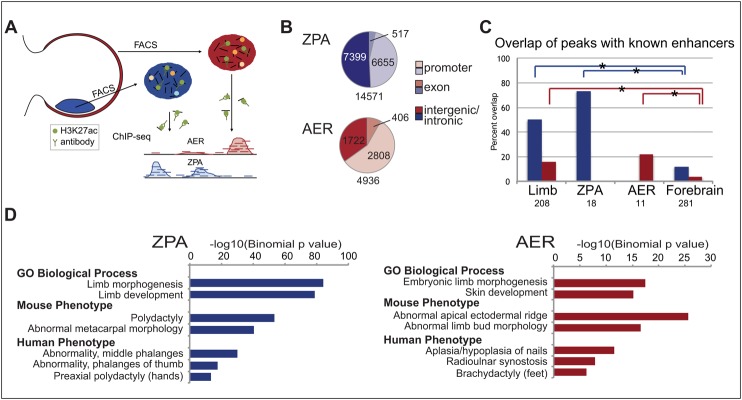
**ChIP-seq from isolated ZPA and AER cells identifies candidate regulatory elements.** (A) Schematic showing cells from the ZPA and AER isolated by FACS, followed by ChIP-seq with an antibody against H3K27ac. (B) Different genomic categories are associated with H3K27ac ZPA and AER peaks. (C) ZPA (blue) and AER (red) ChIP-seq peaks overlap enhancers from the VISTA Enhancer Browser (Visel et al., 2007). Both ZPA and AER peaks show significant overlap with enhancers in the whole limb, but have little overlap with forebrain enhancers (*P<0.0001; Fisher's exact test, one-tailed). ZPA and AER enhancers also overlap more with their respective tissue compared with the forebrain enhancers. (D) GREAT (McLean et al., 2010) shows significantly enriched terms related to ZPA and AER biological function and phenotypes.

We bred transgenic mouse lines that express GFP in the ZPA or AER. For the ZPA, we used *Shh-GFP-cre* mice (Harfe et al., 2004) that have a cassette containing an in-frame fusion between GFP and *cre* inserted at the ATG of the mouse *Shh* gene, leading to specific GFP expression in the ZPA (supplementary material Fig. S1A). For the AER, we crossed *Msx2-cre* mice that expresses *cre* specifically in the AER under the control of the *Msx2* promoter (Sun et al., 2000) to GNZ (ROSA26-nGFP) mice that contain a loxP-flanked STOP cassette followed by a GFP/β-galactosidase fusion protein sequence with an SV40 nuclear localization signal (Stoller et al., 2008), thus allowing for AER-specific GFP expression (supplementary material Fig. S1B). Embryos were harvested at E11.5, and ZPA or AER cells were isolated from dissected limbs by FACS. Similar to previous measurements (Rock et al., 2007), the ZPA and AER cells each comprised 2-4% of the total cells of the limb. These cells were subjected to ChIP-seq with an antibody for H3K27ac (Fig. 1A).

In the ZPA, we identified 14,571 H3K27ac peaks (supplementary material Table S1). Of these, 45% were at promoters [defined as −2500 and +500 bp from the transcription start site (TSS) based on UCSC known gene annotations], 4% in exons (excluding exons that overlap the promoter region) and 51% in intergenic or intronic regions (Fig. 1B). In the AER, we identified 4936 peaks (supplementary material Table S1), 57% of which were located at gene promoters, 8% in exons and 35% in intergenic or intronic regions (Fig. 1B). We next compared these peaks with 208 functionally validated limb enhancers from the VISTA Enhancer Browser (Visel et al., 2007). As a negative control, we used a set of 281 forebrain enhancers with no limb expression. We observed a significant overlap of our peaks with the VISTA limb set versus the forebrain (*P*<0.0001; Fisher's exact test, one-tailed) for both the ZPA and AER (Fig. 1C). Of the 208 VISTA limb enhancers, we identified 11 with ZPA/posterior expression and 18 with AER/ectoderm expression. Our ZPA and AER ChIP-seq peaks overlap more with the validated enhancers in their respective tissue, but the number of enhancers was too low for statistical significance.

**Fig. 2.**
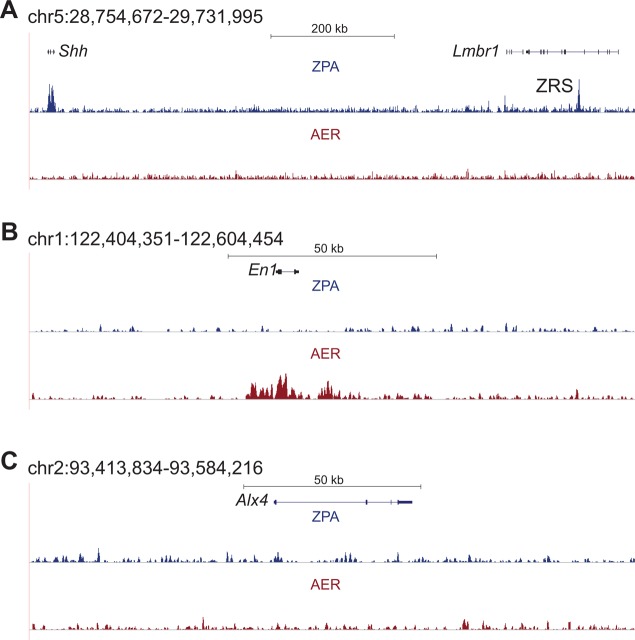
**Genomic regions around limb patterning genes show epigenetic active signatures only in the appropriate tissue.** All coordinates are mm9. (A) The *Shh* gene and its enhancer ZRS are active in the ZPA and overlap H3K27ac. The AER has no peak at this locus. (B) *En1*, an AER- and ventral ectoderm-expressed gene, has an H3K27ac peak in the AER, but not in the ZPA. (C) The locus around *Alx4*, a gene expressed in the anterior limb mesenchyme, does not have an H3K27ac peak in either signaling center.

We used GREAT (McLean et al., 2010) to identify biological functions associated with the ZPA and AER peaks. Numerous limb-associated genes reside near our ChIP-seq peaks (supplementary material Table S2). In both datasets, we observed a significant enrichment for terms related to limb development and malformation phenotypes (Fig. 1D; supplementary material Table S2). The ZPA includes mouse and human phenotypic terms related to polydactyly and abnormal digit patterning, which are common effects of ZPA disruptions. The AER terms include ‘abnormal limb bud morphology’, ‘aplasia of the nails’ and ‘brachydactyly’, all of which are phenotypes associated with AER defects. Limb development and malformation phenotype terms are also enriched when using only non-TSS peaks (supplementary material Fig. S2; Tables S1 and S2), showing that this enrichment is not due to promoter binding. We also examined gene loci known to be highly expressed in the ZPA or AER and observed a correlation with H3K27ac peak presence. As examples, we observed a specific H3K27ac peak for *Shh* and its enhancer ZRS in the ZPA and for engrailed homeobox 1 (*En1*) in the AER, whereas *Alx4*, a gene expressed specifically in the anterior mesenchyme, did not have an H3K27ac signal in either tissue (Fig. 2).

**Fig. 3.**
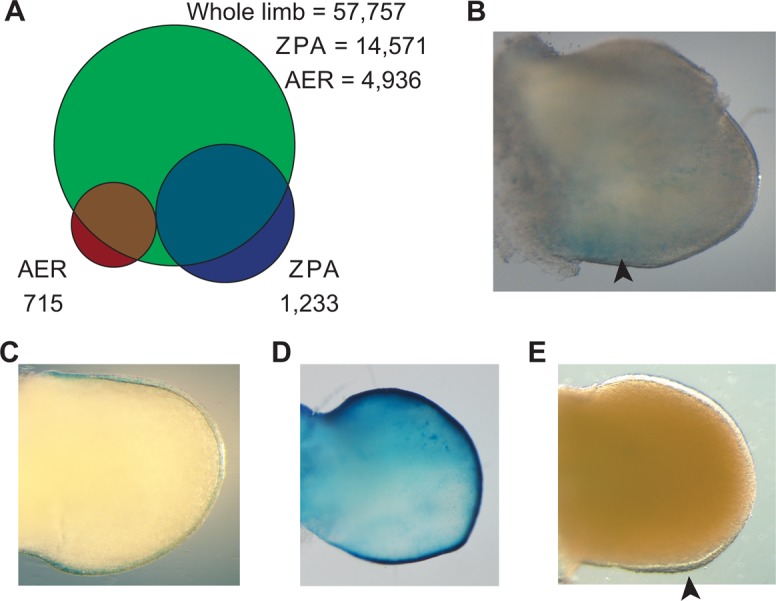
**ZPA and AER specific peak analyses.** (A) By overlapping ZPA and AER H3K27ac peaks to whole-limb H3K27ac peaks, we obtained 1233 ZPA and 715 AER-specific ChIP-seq peaks. (B) ZPA peak 44 shows weak *lacZ* expression in the ZPA in a mouse transgenic enhancer assay. (C-E) AER peaks 2292, 3723 and 4460 show AER limb expression in a mouse transgenic assay. Arrowheads highlight β-galactosidase-stained regions.

To identify ZPA and AER-specific enhancers, we selected peaks that did not overlap with the promoters of known genes or with previously published E11.5 whole-limb H3K27ac and p300 ChIP-seq datasets (Cotney et al., 2012; Visel et al., 2009). The resulting lists contain 1233 ZPA-specific and 715 AER-specific peaks (Fig. 3A). To functionally validate these peaks, we tested five candidates from each signaling center for enhancer activity in mice, prioritizing enhancers near genes involved in limb development. These genes include transcription factors, signal receptors and genes involved in cilia transport that are required for *Shh* signaling (supplementary material Table S3).

Of the five ZPA candidates, one (ZPA44) showed weak enhancer expression in the posterior limb bud (Fig. 3B). ZPA44 is located 30 kb upstream of *Tcfap2b* (*Tfap2b* – Mouse Genome Informatics), a gene expressed in the ZPA at levels nearly four times higher than in the rest of the limb (Rock et al., 2007). Out of the five AER candidates, three were positive for enhancer activity in the AER and ectoderm layer of the limb bud (Fig. 3C-E). AER2292 is located 600 kb upstream of *Fgfr2*, which is necessary for limb bud development (Xu et al., 1998). AER3723 is 142 kb downstream of *Sp8*, a transcription factor expressed in the AER required for limb bud outgrowth (Bell et al., 2003). The third AER enhancer, AER4460, showed weak posterior AER staining and is located in the first intron of the protein S gene (*Pros1*), 15 kb upstream of ADP-ribosylation factor-like 13b (*Arl13b*), which is involved in the localization of *Shh* receptor pathway components in the cilium (Larkins et al., 2011).

## DISCUSSION

We believe that these datasets will be valuable to researchers studying evolutionary changes in limb morphology and human congenital limb malformations. It has previously been demonstrated that replacing the mouse *Prx1* limb enhancer with the bat homologous enhancer can lead to longer bones (Cretekos et al., 2008), and our dataset of ZPA and AER enhancers can provide a unique resource to identify additional limb enhancers that could have led to limb morphological differences. Human limb malformations are the second-most common form of birth defect, with a frequency of nearly 1 in 500 live births (Moore and Persaud, 1998). Some of these occur as part of a syndrome; however, limb defects are also seen in isolation as the only phenotype in an individual and have been found to be caused by enhancer mutations (Dathe et al., 2009; Kurth et al., 2009; Lettice et al., 2003). An enhancer that regulates the ZPA or AER could be a site where mutations result in limb defects. Our ZPA and AER ChIP-seq peaks thus provide specific candidate sequences in the genome that, when mutated, can lead to morphological differences between species and limb malformations and can be used to guide mutation analyses between organisms and in individuals with isolated limb malformations.

Previous whole-limb approaches have failed to identify enhancers that are active in these important limb development signaling centers, as seen by *in vivo* results (Visel et al., 2009), possibly because the cells of these regions comprise only a small proportion of the limb tissue. By investigating these specific tissues, our study provides a novel resource of putative ZPA and AER enhancers. The rate of *in vivo* enhancer validation for this dataset was low, particularly in the ZPA. This could be due to the stringency of our peak selection. By eliminating peaks in our dataset that had any overlap to a region called as a peak in the whole-limb set, we probably eliminated enhancers that are biologically specific to the ZPA or AER, but produced a signal that was identifiable in the whole-limb set. These appear to include some of the strongest enhancers because we find that the distribution of peak heights in the signaling center-specific sets is lower than the distribution for the entire set of either the ZPA or AER (supplementary material Fig. S3). For example, the ZRS, which is known to control ZPA-specific expression of *Shh*, is called as a peak in the whole-limb H3K27ac dataset under some parameters (Cotney et al., 2012).

In summary, using FACS followed by low-cell ChIP-seq on ZPA and AER cells, we identified thousands of potential limb signaling center peaks. We observed a strong correlation between known ZPA- and AER-specific gene loci and H3K27ac peak prevalence. We also observed a strong correlation with fitting biological terms and limb-associated genes using GREAT (McLean et al., 2010) (supplementary material Table S2). These ZPA- and AER-specific regions represent a set of enhancers active in regions that determine limb growth and patterning and can help inform our understanding of tetrapod limb development, evolutionary differences in limb morphology and the genetic etiology of human limb malformations.

## MATERIALS AND METHODS

### Tissue collection

All mouse work was approved by the UCSF institutional animal care and use committee.

To isolate cells from the ZPA and AER, transgenic mouse lines were established that express GFP in one of these regions. For the ZPA, we used the *Shh*^tm1(EGFP/cre)Cjt^ line (Harfe et al., 2004). Heterozygous males were mated to CD-1 females and GFP-positive embryos were used for tissue collection. For the AER, homozygous male tg(*Msx2*-cre)5Rem mice (Sun et al., 2000) were mated to females homozygous for a floxed reporter B6.129-GT(ROSA)26Sor^tm1Joe^ (Stoller et al., 2008). All mice were backcrossed onto a CD-1 strain to address any variation of embryo size between lines.

Embryos were collected at E11.5 from timed matings. For the ZPA, embryos were examined briefly with a dissecting fluorescent microscope to select those carrying the GFP allele. Both forelimbs and hindlimbs were collected and crosslinked with 1% formaldehyde for 10 min at room temperature, followed by quenching with glycine and rinsing with cold PBS. Tissue was homogenized with a Dounce tissue grinder (Kontes) to a single-cell suspension for FACS. Cells were kept on ice in 0.5 μM EDTA and 0.05% BSA in PBS for sorting as previously described (Rock et al., 2007) and sorted on a FACSAria II (BD Biosciences). GFP-positive cells represented ∼2-4% of the limb tissue for both ZPA and AER. Cells were collected in the sorting buffer, pelleted by centrifugation and flash-frozen to −80°C.

### ChIP-seq

ChIP was performed using the LowCell#ChIP kit (Diagenode) with modified wash conditions (see methods in the supplementary material). Cells from dissected limbs from an estimated 20-35 embryos were pooled to give ∼70,000 cells per IP; they were sonicated with a Covaris sonicator (S220 Focused-ultrasonicator, Covaris), and 30 µl of sheared chromatin was used for each ChIP. The antibody anti-acetyl histone H3 (Lys27) clone CMA309 (Millipore, 05-1334; 4 µg per IP) was used. Libraries were constructed using a pre-release beta version of the Rubicon ThruPLEX (now ThruPLEX-FD, Rubicon Genomics) library construction kit according to the manufacturer's directions. For each library, 10 µl of ChIP material was used with a total of 14 cycles of amplification.

Sequencing on an Illumina HiSeq was carried out at the UC Davis Genome Center, and FASTQ files were aligned to the *Mus musculus* genome (UCSC build mm9) using Bowtie 0.12.8 (Langmead et al., 2009). A single bp mismatch was permitted and reads with multiple alignments were discarded. From 43,109,366 and 40,996,716 reads for the AER and ZPA samples, respectively, 31,258,376 and 30,757,898 aligned uniquely. The input sample was sequenced more deeply, with 111,754,456 reads total and 80,925,525 aligning uniquely. In each case, ∼11% of sequences failed to align, and 15% of were suppressed due to multiple alignments.

The alignments were sorted and indexed using SAMtools 0.1.18 (Li et al., 2009) and converted to BED files using the utility bam2bed, a part of bedtools 2.17.0 (Quinlan and Hall, 2010). The peak-finding tool SICER 1.1 (Zang et al., 2009) was used to identify enriched H3K27ac islands in the AER and ZPA samples, with the input sample as the control library. The settings used were as follows: redundancy threshold=1, window size=200 bp, fragment size=300 bp, effective genome fraction=0.74, gap size=200 bp, FDR=0.01. For our GREAT (McLean et al., 2010) analysis, we used the Basal Plus Extension setting with proximal 5 kb upstream and 1 kb downstream and distal 1 Mb extension.

### Mouse transgenic enhancer assays

Ten selected peaks were PCR-amplified from mouse genomic DNA. Coordinates of peaks and primer sequences are available in supplementary material Table S3. The enhancer candidates were cloned as individual sequences into an Hsp68-*lacZ* enhancer assay vector (Kothary et al., 1989) using the Gateway system (Life Technologies). Transgenic mouse assays followed by β-galactosidase staining were carried out through Cyagen Biosciences using standard procedures (Nagy et al., 2002; Pennacchio et al., 2006) and embryos were examined at mouse E11.5.

### Data access

ChIP-seq data from this study is available in SRA (http://www.ncbi.nlm.nih.gov/sra) (SRA experiment: SRX597164; AER: SRR1393727; ZPA: SRR1393728).

## Supplementary Material

Supplementary Material

## References

[DEV110965C1] Anderson E., Peluso S., Lettice L. A. and Hill R. E. (2012). Human limb abnormalities caused by disruption of hedgehog signaling. *Trends Genet.* 28, 364-373 10.1016/j.tig.2012.03.012 22534646

[DEV110965C2] Bell S. M., Schreiner C. M., Waclaw R. R., Campbell K., Potter S. S. and Scott W. J. (2003). Sp8 is crucial for limb outgrowth and neuropore closure. *Proc. Natl. Acad. Sci. USA* 100, 12195-12200 10.1073/pnas.2134310100 14526104 PMC218735

[DEV110965C3] Cotney J., Leng J., Oh S., DeMare L. E., Reilly S. K., Gerstein M. B. and Noonan J. P. (2012). Chromatin state signatures associated with tissue-specific gene expression and enhancer activity in the embryonic limb. *Genome Res.* 22, 1069-1080 10.1101/gr.129817.111 22421546 PMC3371702

[DEV110965C4] Cotney J., Leng J., Yin J., Reilly S. K., DeMare L. E., Emera D., Ayoub A. E., Rakic P. and Noonan J. P. (2013). The evolution of lineage-specific regulatory activities in the human embryonic limb. *Cell* 154, 185-196 10.1016/j.cell.2013.05.056 23827682 PMC3785101

[DEV110965C5] Cretekos C. J., Wang Y., Green E. D., Martin J. F., Rasweiler J. J. and Behringer R. R. (2008). Regulatory divergence modifies limb length between mammals. *Genes Dev.* 22, 141-151 10.1101/gad.1620408 18198333 PMC2192750

[DEV110965C6] Creyghton M. P., Cheng A. W., Welstead G. G., Kooistra T., Carey B. W., Steine E. J., Hanna J., Lodato M. A., Frampton G. M., Sharp P. A. et al. (2010). Histone H3K27ac separates active from poised enhancers and predicts developmental state. *Proc. Natl. Acad. Sci. USA* 107, 21931-21936 10.1073/pnas.1016071107 21106759 PMC3003124

[DEV110965C7] Dathe K., Kjaer K. W., Brehm A., Meinecke P., Nürnberg P., Neto J. C., Brunoni D., Tommerup N., Ott C. E., Klopocki E. et al. (2009). Duplications involving a conserved regulatory element downstream of BMP2 are associated with brachydactyly type A2. *Am. J. Hum. Genet.* 84, 483-492 10.1016/j.ajhg.2009.03.001 19327734 PMC2667973

[DEV110965C8] Harfe B. D., Scherz P. J., Nissim S., Tian H., McMahon A. P. and Tabin C. J. (2004). Evidence for an Expansion-based temporal SHH gradient in specifying vertebrate digit identities. *Cell* 118, 517-528 10.1016/j.cell.2004.07.024 15315763

[DEV110965C9] Kothary R., Clapoff S., Darling S., Perry M. D., Moran L. A. and Rossant J. (1989). Inducible expression of an hsp68-lacZ hybrid gene in transgenic mice. *Development* 105, 707-714. 2557196 10.1242/dev.105.4.707

[DEV110965C10] Kurth I., Klopocki E., Stricker S., van Oosterwijk J., Vanek S., Altmann J., Santos H. G., van Harssel J. J. T., de Ravel T., Wilkie A. O. M. et al. (2009). Duplications of noncoding elements 5′ of SOX9 are associated with brachydactyly-anonychia. *Nat. Genet.* 41, 862-863 10.1038/ng0809-862 19639023

[DEV110965C11] Langmead B., Trapnell C., Pop M. and Salzberg S. L. (2009). Ultrafast and memory-efficient alignment of short DNA sequences to the human genome. *Genome Biol.* 10, R25 10.1186/gb-2009-10-3-r25 19261174 PMC2690996

[DEV110965C12] Larkins C. E., Aviles G. D. G., East M. P., Kahn R. A. and Caspary T. (2011). Arl13b regulates ciliogenesis and the dynamic localization of Shh signaling proteins. *Mol. Biol. Cell* 22, 4694-4703 10.1091/mbc.E10-12-0994 21976698 PMC3226485

[DEV110965C13] Lettice L. A., Heaney S. J. H., Purdie L. A., Li L., de Beer P., Oostra B. A., Goode D., Elgar G., Hill R. E. and de Graaff E. (2003). A long-range Shh enhancer regulates expression in the developing limb and fin and is associated with preaxial polydactyly. *Hum. Mol. Genet.* 12, 1725-1735 10.1093/hmg/ddg180 12837695

[DEV110965C14] Li H., Handsaker B., Wysoker A., Fennell T., Ruan J., Homer N., Marth G., Abecasis G. and Durbin R. (2009). The sequence alignment/map format and SAMtools. *Bioinformatics* 25, 2078-2079 10.1093/bioinformatics/btp352 19505943 PMC2723002

[DEV110965C15] McLean C. Y., Bristor D., Hiller M., Clarke S. L., Schaar B. T., Lowe C. B., Wenger A. M. and Bejerano G. (2010). GREAT improves functional interpretation of cis-regulatory regions. *Nat. Biotechnol.* 28, 495-501 10.1038/nbt.1630 20436461 PMC4840234

[DEV110965C16] Moore K. L. and Persaud T. V. N. (1998). *The Developing Human: Clinically Oriented Embryology*, 6th edn Philadelphia: Saunders.

[DEV110965C17] Nagy A., Gertsenstein M., Vintersten K. and Behringer R. (2002). *Manipulating the Mouse Embryo: A Laboratory Manual*, 3rd edn Cold Spring Harbor, NY: Cold Spring Harbor Laboratory Press.

[DEV110965C18] Pennacchio L. A., Ahituv N., Moses A. M., Prabhakar S., Nobrega M. A., Shoukry M., Minovitsky S., Dubchak I., Holt A., Lewis K. D. et al. (2006). In vivo enhancer analysis of human conserved non-coding sequences. *Nature* 444, 499-502 10.1038/nature05295 17086198

[DEV110965C19] Quinlan A. R. and Hall I. M. (2010). BEDTools: a flexible suite of utilities for comparing genomic features. *Bioinformatics* 26, 841-842 10.1093/bioinformatics/btq033 20110278 PMC2832824

[DEV110965C20] Rada-Iglesias A., Bajpai R., Prescott S., Brugmann S. A., Swigut T. and Wysocka J. (2012). Epigenomic annotation of enhancers predicts transcriptional regulators of human neural crest. *Cell Stem Cell* 11, 633-648 10.1016/j.stem.2012.07.006 22981823 PMC3751405

[DEV110965C21] Rock J. R., Lopez M. C., Baker H. V. and Harfe B. D. (2007). Identification of genes expressed in the mouse limb using a novel ZPA microarray approach. *Gene Expr. Patterns* 8, 19-26 10.1016/j.modgep.2007.08.004 17911046

[DEV110965C22] Stoller J. Z., Degenhardt K. R., Huang L., Zhou D. D., Lu M. M. and Epstein J. A. (2008). Cre reporter mouse expressing a nuclear localized fusion of GFP and β-galactosidase reveals new derivatives of Pax3-expressing precursors. *Genesis* 46, 200-204 10.1002/dvg.20384 18395835 PMC2747029

[DEV110965C23] Sun X., Lewandoski M., Meyers E. N., Liu Y.-H., Maxson R. E. and Martin G. R. (2000). Conditional inactivation of Fgf4 reveals complexity of signalling during limb bud development. *Nat. Genet.* 25, 83-86 10.1038/75644 10802662

[DEV110965C24] VanderMeer J. E. and Ahituv N. (2011). cis-regulatory mutations are a genetic cause of human limb malformations. *Dev. Dyn.* 240, 920-930 10.1002/dvdy.22535 21509892 PMC3174732

[DEV110965C25] Visel A., Minovitsky S., Dubchak I. and Pennacchio L. A. (2007). VISTA Enhancer Browser--a database of tissue-specific human enhancers. *Nucleic Acids Res.* 35, D88-D92 10.1093/nar/gkl822 17130149 PMC1716724

[DEV110965C26] Visel A., Blow M. J., Li Z., Zhang T., Akiyama J. A., Holt A., Plajzer-Frick I., Shoukry M., Wright C., Chen F. et al. (2009). ChIP-seq accurately predicts tissue-specific activity of enhancers. *Nature* 457, 854-858 10.1038/nature07730 19212405 PMC2745234

[DEV110965C27] Xu X., Weinstein M., Li C., Naski M., Cohen R. I., Ornitz D. M., Leder P. and Deng C. (1998). Fibroblast growth factor receptor 2 (FGFR2)-mediated reciprocal regulation loop between FGF8 and FGF10 is essential for limb induction. *Development* 125, 753-765. 9435295 10.1242/dev.125.4.753

[DEV110965C28] Zang C., Schones D. E., Zeng C., Cui K., Zhao K. and Peng W. (2009). A clustering approach for identification of enriched domains from histone modification ChIP-Seq data. *Bioinformatics* 25, 1952-1958 10.1093/bioinformatics/btp340 19505939 PMC2732366

[DEV110965C29] Zeller R., López-Ríos J. and Zuniga A. (2009). Vertebrate limb bud development: moving towards integrative analysis of organogenesis. *Nat. Rev. Genet.* 10, 845-858 10.1038/nrg2681 19920852

