# Supplementary material for: Genome-wide identification of signaling center enhancers in the developing limb
Source: Development. 2014 Nov;141(21):4194–8. doi: 10.1242/dev.110965 (PMC4302890; doi:10.1242/dev.110965)
Supplement: Supplementary Material [file supp_141_21_4194__index.html]

Supplementary Material 

# Genome-wide identification of signaling center enhancers in the developing limb

## DEV110965 Supplementary Material

**Files in this Data Supplement:**

- Supplementary Material
