# Supplementary material for: Genome-wide identification of signaling center enhancers in the developing limb
Source: Development. 2014 Nov;141(21):4194–8. doi: 10.1242/dev.110965 (PMC4302890; doi:10.1242/dev.110965)
Supplement: Supplementary Material [file supp_dev.110965_DEV110965supp.pdf]

**Supplementary materials and methods**

*ChIP washes*

After incubating the chromatin with antibody-coated beads overnight at $4^{o}$ C, the beads were washed three times with 125ul of ice cold Buffer A. All washes were performed in a $4^{o}$ C cold room as follows: Add buffer, close cap and invert to resuspend beads, incubate on rotating wheel for 5 minutes, inverting by hand and make sure beads remain suspended at 2 minutes and 4 minutes, spin, collect beads on magnetic rack for 2 minutes, remove buffer.

*Visualizing ChIP-seq data*

To produce coverage pileups, AER and ZPA reads were extended to the predicted fragment size of 300bp using the slopBed utility in bedtools. A BED file encompassing the mm9 genome in 100bp intervals was generated, and overlapping AER, ZPA and input reads were counted in each interval using the bedtools utility coverageBed. The counts were then normalized by dividing by the total number of aligned reads for the respective sample, after which the normalized input signal was subtracted off the normalized AER and ZPA signals. The AER and ZPA signals were then scaled by a factor of 80,925,525 (the # of input reads) to facilitate viewing.

## Supplementary Figures

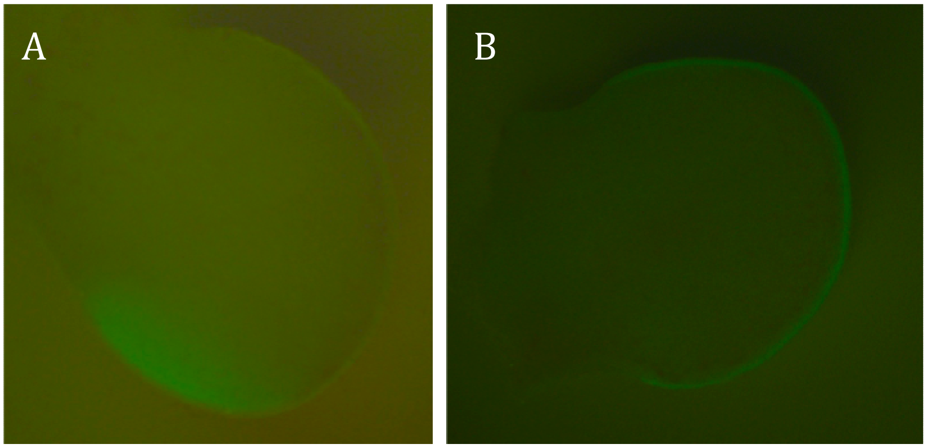


**Fig. S1.** ZPA- and AER-specific GFP expression. (*A*) A limb from E11.5 Shh$^{tm1(EGFP/cre)Cjt}$ mice showing ZPA specific expression. (*B*) An E11.5 limb from a homozygous male mice carrying the Cre transgene (tg(Msx2-cre) crossed to a floxed reporter (B6.129-GT(ROSA)26Sor$^{tm1Joe}$/J) showing specific GFP expression in the AER.

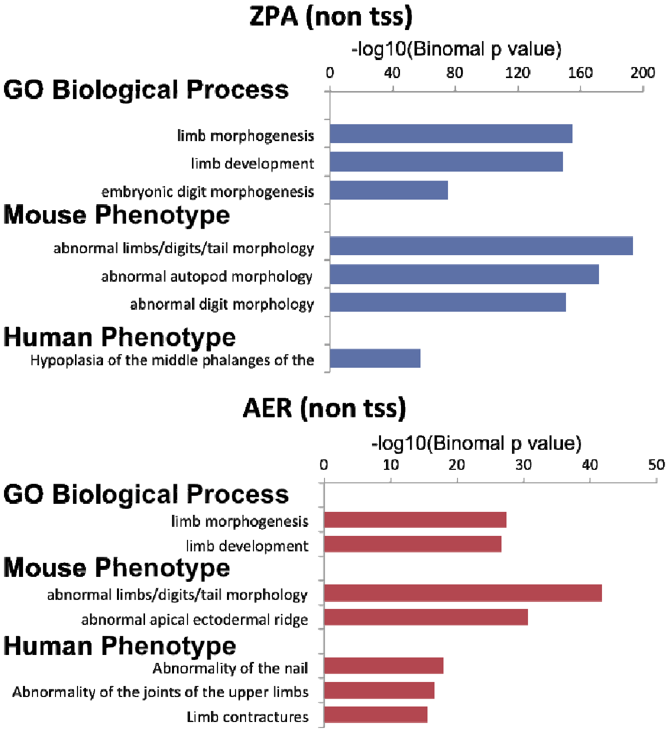


**Fig. S2.** GREAT results for non-TSS peaks from the ZPA and AER ChIP-seq datasets show GO terms and phenotypes associated with the roles of the ZPA and AER in limb development.

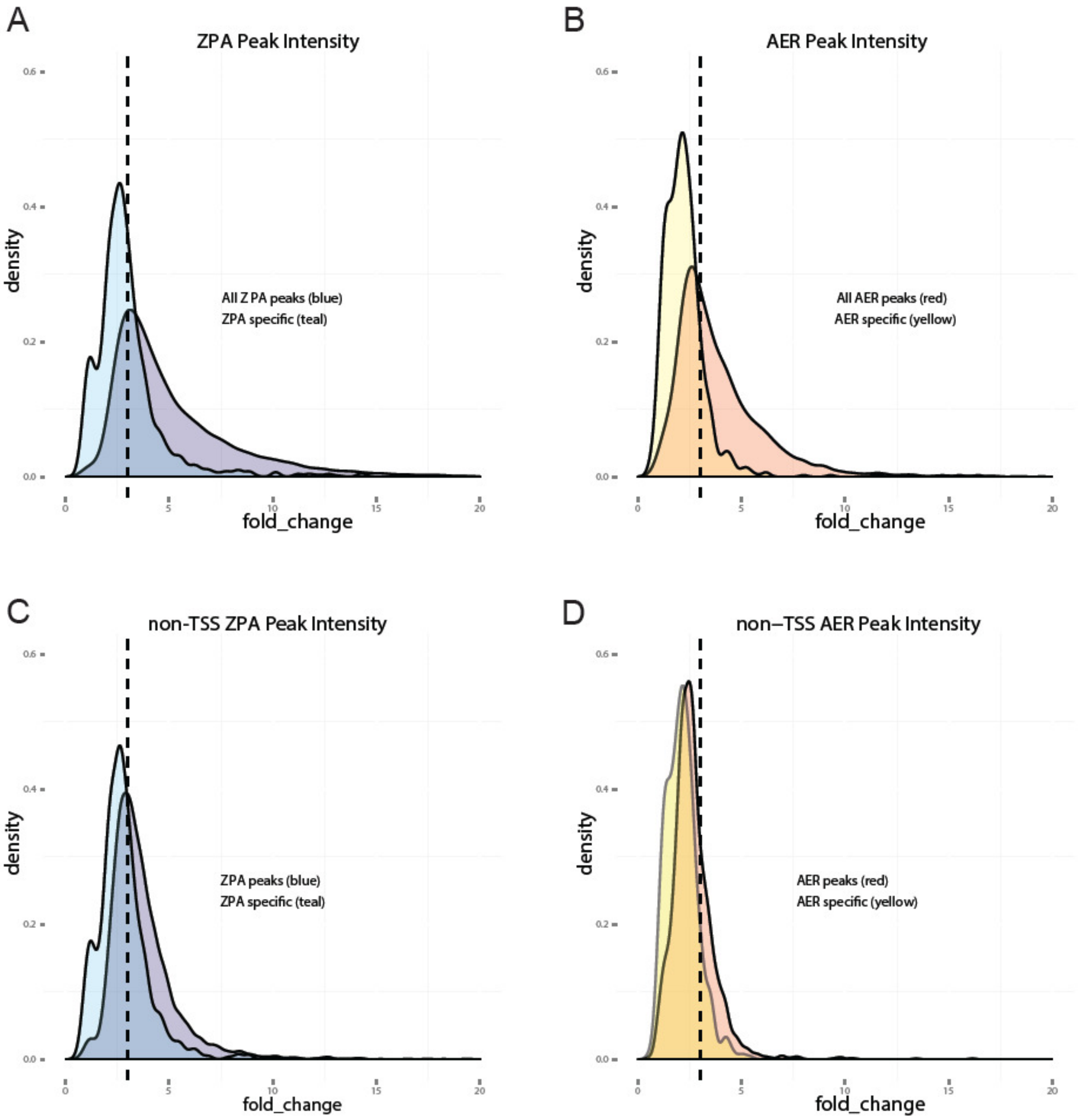


**Fig. S3.** Peak height and signaling center specificity. The distribution of peak signal intensity shows that the ZPA and AER-specific peaks have a weaker signal in general compared to the entire ZPA and AER sets. (A) The distribution of all ZPA peaks (blue) compared to ZPA specific peaks (peaks with no overlap to whole limb) (teal) shows that the peaks with no overlap are generally weaker. (B) The distribution of all AER peaks (red) compared to AER specific peaks (peaks with no overlap to whole limb) (orange) shows that the peaks with no overlap are generally weaker. (C,D) The same trend is seen when looking only at non-TSS peaks from either data set. This suggests that some of the very strongest enhancer signals are the ones that overlap with whole-limb signal.

## Supplementary Tables

Table S1

Click here to Download Table S1

Table S2

Click here to Download Table S2

**Table S3.** Primers for mouse transgenic assays

| Peak | Coordinates (mm9) | PrimerF | PrimerR | Limb gene (location) |
|---|---|---|---|---|
| AER203 | chr1:122510000-122512399 | GTAGGAGGAGGCTTCATTGC | CTCCAGCTCGCCATACACTA | En1 (+12136) |
| AER2292 | chr7:136794000-136796399 | CCAAGAGCATATCCCCATCT | CAGTGATAGACAGCCCTGGA | Fgfr2 (+615122) |
| AER3723 | chr12:119942200-119943599 | AGAGAGAGAGAGCACCTCATCAA | GACATCTGGGCTCACTTCCT | Sp8 (-141902) |
| AER308 | chr1:170693400-170694599 | GGCCGACCTGTTACTTGTTT | GGGGATAGCAGGCTACTCAG | Pbx1 (-331611) |
| AER4460 | chr16:62861400-62862199 | TGCCATTTTATTGCAAGTCAG | TCAGCAGATTATTCGTAAGTGG | Arl13b (+15345) |
| ZPA6706 | chr8:60898600-60899199 | CATGGTTATTTTGTTCCACATTTT | GCAATGCTTTTCTTTTGTGG | Hand2 (-1098890) |
| ZPA12651 | chr16:49332600-49333199 | TTTAAAAATATTGAAAAGGTGGCTA | GCAGTAGACCTTAGGGGAGCA | Ift57 (-366507) |
| ZPA1563 | chr2:93377800-93378599 | GCTCGGTACTTGCTCAGGAC | CTCTCTCCCCAAATCCCTTC | Alx4 (-104391) |
| ZPA2794 | chr3:130665200-130665799 | TACCAAGCACTTACTACAGGCTACA | ATAGGGGCGGTCCTTTTATT | Lef1 (-147889) |
| ZPA44 | chr1:19231200-19231999 | CCTGTGGGTCTTCTCCTTTG | CATCCTGTCACCAACCCTCT | Tcfap2b (+29465) |
